# Bta-miR-199a-3p Inhibits LPS-Induced Inflammation in Bovine Mammary Epithelial Cells via the PI3K/AKT/NF-κB Signaling Pathway

**DOI:** 10.3390/cells11213518

**Published:** 2022-11-07

**Authors:** Yuhang Li, Qianqian Ren, Xingping Wang, Zhuoma Luoreng, Dawei Wei

**Affiliations:** 1 School of Agriculture, Ningxia University, Yinchuan 750021, China; 2 Key Laboratory of Ruminant Molecular Cell Breeding, Ningxia Hui Autonomous Region, Yinchuan 750021, China

**Keywords:** dairy cow, bta-miR-199a-3p, mastitis, mammary epithelial cells, inflammation

## Abstract

Mastitis is characterized by inflammatory damage to mammary gland tissue, which could decline milk production and quality and significantly affect the economic benefits of ranching. MicroRNAs (miRNAs), such as miR-199a-3p, are novel therapeutic targets in inflammation, and their regulation is an effective strategy for inflammation control. Despite its importance in humans and animals, the molecular mechanism of bovine miR-199a-3p (bta-miR-199a-3p) in dairy cow mastitis and bovine mammary epithelial cell (bMEC) inflammation is unclear. In our study, a bovine mammary epithelial cell line (MAC-T) induced by lipopolysaccharide (LPS) was used as an inflammatory cell model to investigate the molecular mechanism of bta-miR-199a-3p in the MAC-T inflammatory response. bta-miR-199a-3p was up-regulated in the LPS-induced MAC-T cells, while CD2-associated protein (CD2AP) was revealed as its target gene in a double luciferase reporter gene experiment. In addition, the overexpression of bta-miR-199a-3p negatively regulated the expression of CD2AP and the activation of the phosphatidylinositol 3-kinase (PI3K)/AKT/nuclear factor kappa-B (NF-κB) signaling pathway. These subsequently inhibited the secretion of related inflammatory factors (TNF-α, IL-1β, and IL-6) and the expression of apoptotic genes (*CASP3* and *CASP9*), thereby alleviating the LPS-challenged inflammatory response in the MAC-T cells. Silencing of bta-miR-199a-3p, however, reversed the above effects. Thus, bta-miR-199a-3p inhibits LPS-induced inflammation in bMECs by directly targeting CD2AP and regulating the PI3K/AKT/NF-κB signaling pathway. This study reveals the potential regulatory mechanism of bta-miR-199a-3p in bMEC inflammatory immune response and may serve as a useful target for the treatment of mastitis.

## 1. Introduction

Inflammation is the most common pathological process in various body tissues and organs. Mastitis is a common disease affecting the yield and quality of cow milk [1]. Without effective treatment, mastitis causes decreased fecundity and reduced the life span of dairy cows, leading to numerous economic losses [2]. The etiology of mastitis is complex and is related to several factors, such as genetics, pathogenic microbial infections, and feeding management [3]. Among these, genetic factors are closely associated with susceptibility and resistance to mastitis [4]. Several genes also regulate the progression of mastitis in dairy cows [5,6]. However, the molecular mechanisms are not fully elucidated. *Escherichia coli* is one of the most common bacteria causing clinical mastitis. It usually attacks mammary glands in the early stage of lactation, and sometimes has fatal consequences if not treated in time [7]. Lipopolysaccharide (LPS), the main component of *E. coli* outer membrane, promotes the secretion of tumor necrosis factor-α (TNF-α), interleukin-1β (IL-1β) and interleukin-6 (IL-6), and other inflammatory cytokines [8], and activates various signaling pathways, which are the main factors for initiating inflammatory reactions and body immunity [3,9]. Bovine mammary epithelial cells (bMECs) are the initial lines of protection against the infection by pathogenic microorganisms in the tissues and basal cells of the dairy cow mammary gland immune function [10]. After LPS induction, bovine mammary epithelial cell line (MAC-T) is often used as the cell model in dairy cow mastitis research [11].

MicroRNAs (miRNAs) are endogenous small non-coding RNAs, and widely exist in eukaryotic cells. miRNAs regulate the transcription and translation of mRNAs by targeting their 3′-untranslated region (3′-UTR). They are also involved in the regulation of various physiological and pathological processes [12] and play an indispensable role in various inflammatory and cancer diseases [13]. Several studies have screened and found many differentially expressed miRNAs in cow mastitis, but only a few have been studied functionally [14]. Many studies have also pointed out that miR-199a-3p serves an indispensable molecular regulatory role in several cancers [15,16].

In addition, only a few articles have reported the role that miR-199a-3p plays in inflammation. For example, Peng et al. [17] found that miR-199a-3p attenuated cervical epithelial cell inflammation in mice during preterm labor by targeting high-mobility group box 1 protein (HMGB1) through the TLR4/NF-κB pathway. In mouse ulcerative colitis, miR-199a-3p improves the intestinal barrier by down-regulating the IL-17A/IL-23 axis [18]. In humans, miR-199a-3p decreases IKKβ protein expression, NF-κB activity, and IL-8 secretion in cystic fibrosis cells, thereby reducing chronic lung inflammation [19]. miR-199a-3p also regulates inflammatory diseases in humans and mice through some mRNA and related signaling pathways. However, no regulation of bovine miR-199a-3p (bta-miR-199a-3p) has been reported in dairy cow mastitis. To explore the regulatory role of bta-miR-199a-3p in dairy cow mastitis, the target gene of the miRNA was identified, and its role in LPS-induced inflammation of MAC-T cells and its molecular mechanism were studied. The findings of our study will provide a novel direction for analyzing the molecular regulatory network of dairy cow mastitis and searching for molecular therapeutic targets.

## 2. Materials and Methods

### 2.1. Cell Culture

293T cells were thawed and cultured in Dulbecco’s modified eagle medium (DMEM) (Hyclone, Logan, UT, USA) supplemented with 5% fetal bovine serum (FBS) (System Biosciences, Mountain View, CA, USA). Laboratory-identified frozen MAC-T cells were thawed and cultured in DMEM/F12 (Hyclone, Logan, UT, USA) containing 10% FBS [11,20] (Figure S1). The 293T cells and MAC-T cells were maintained at 37 °C, in 5% CO_2_ concentration, and at 100% saturated humidity.

### 2.2. Inflammation Induction of MAC-T Cells

The cultured MAC-T cells were inoculated in 6-well plates and then treated with 50 ng/μL of LPS to induce inflammation [21] after fusing from 70% to 80%. The cells were collected at 0 (control), or 3, 6, 12, and 24 h (induced), for RNA extraction and detecting bta-miR-199a-3p expression.

### 2.3. MAC-T Cell Transfection

The MAC-T cells were inoculated in 6-well plates. When the density of the cells was about 60–70%, bta-miR-199a-3p mimic, mimic NC, bta-miR-199a-3p inhibitor, or inhibitor NC were transiently transfected into the cells using the X-Tremene HP DNA transfection reagent (Roche, Basel, Switzerland) in accordance with the manufacturer’s instructions, with three replicates for each transfection group. The transfection efficiency was observed using an inverted fluorescence microscope (Olympus Corporation, Tokyo, Japan). The overexpression or inhibition efficiency was detected using quantitative real-time PCR (qRT-PCR). The bta-miR-199a-3p mimic, mimic NC, bta-miR-199a-3p inhibitor, and inhibitor NC were designed and synthesized by Ribobio Co., Guangzhou, China. A 50 ng/μL of LPS was added 42 h after transfection to induce inflammation. The cells were harvested 6 h later for subsequent RNA extraction and detection of related mRNA and protein expression.

### 2.4. Target Gene Prediction

The sequence of bta-miR-199a-3p was obtained from the miRBase database (http://www.mirbase.org/ (accessed on 5 January 2021)). Two kinds of miRNA target gene prediction software, TargetScan (http://www.targetscan.org/vert_72/ (accessed on 5 January 2021)) and miRWalk (http://mirwalk.umm.uni-heidelberg.de/ (accessed on 5 January 2021)), were used to predict the target genes of bta-miR-199a-3p. The VENNY 2.1 online software (https://bioinfogp.cnb.csic.es/tools/venny/ (accessed on 5 January 2021)) was used to take the intersection to finally determine the potential target gene of bta-miR-199a-3p.

### 2.5. Construction of Recombinant Plasmid of Double Luciferase Reporter Gene and Identification of Target Gene of Bta-miR-199a-3p

The 3′-UTR of the bovine CD2AP and MTOR mRNA containing the potential binding site of bta-miR-199a-3p was amplified using PCR with specific primers (Table S1). The restriction endonucleases Xho I and Not I were used to construct a wild-type 3′UTR-wt-CHECK^TM^-2 double luciferase reporter gene vector. To verify the binding sites of bta-miR-199a-3p and CD2AP, we commissioned a biological company (Sangon Biotech, Shanghai, China) to chemically synthesize a vector with mutations (mut) at the potential binding site of CD2AP and to construct a 3′UTR-mut-CHECK^TM^-2 mutant vector. The reliability of all constructs was verified by sequencing.

When the 293T cells’ density reached 60–70% in 24-well plates, the 3′UTR-wt-CHECK^TM^-2 (or 3′UTR-mut-CHECK^TM^-2) recombinant plasmid was co-transfected with bta-miR-199a-3p mimic into the 293T cells using an X-Tremene HP DNA transfection reagent, and a mimic NC control group was established. The Dual-Luciferase^®^ Reporter Assay System was used to perform luciferase activity analysis in strict accordance with the manufacturer’s instructions (Promega, Madison, WI, USA). The ratio of renilla luciferase activity to firefly luciferase activity significantly decreased in the 3′UTR-wt-CHECK^TM^-2+mimic group (*p* < 0.05), demonstrating that the miRNA had a targeted binding relationship with the corresponding mRNA.

### 2.6. RNA Extraction and qRT-PCR

Total RNA was extracted from the MAC-T cells using the TriZol kit according to the manufacturer’s instructions (Takara, Beijing, China). RNA quality and concentration were determined using electrophoresis and a Multi-Mode Reader (BioTek, Winooski, VA, USA). The Primer Premier 5.0 (Premier Biosoft, Palo Alto, CA, USA) software was used to design stem ring reverse transcription primer for bta-miR-199a-3p (RT: 5′-GTCGTATCCAGTGCGTGTCGTGGAGTCGGCAATTGCACTGGATACGACTAACCAAT-3′) and qRT-PCR primers for its related genes (Table S2). The cDNA synthesis of bta-miR-199a-3p and related mRNA was performed using the PrimeScript^TM^ RT Reagent Kit with gDNA Eraser (Takara, Beijing, China). Using this cDNA as a template, the expression levels of bta-miR-199a-3p and related mRNA in the cells were detected using a CFX-96 Touch Real-Time PCR instrument (BioRad, Hercules, CA, USA) and 2 × M5 HiPer SYBR Premix EsTaq (with Tli RNaseH) (Mei5bio, Beijing, China) [22]. The qRT-PCR reaction was carried out in a 20 μL PCR mix consisting of 10 μL of 2 × M5 HiPer SYBR Premix EsTaq (with Tli RNaseH), 0.8 μL of each of the upstream and downstream primers, and 1.0 μL of cDNA (100 ng/μL). The PCR reactions were performed as follows: pre-denaturation at 95 °C for 30 s, at 95 °C for 5 s, and at 60 °C for 30 s for 40 cycles. The genes of glyceraldehyde 3-phosphate dehydrogenase (*GAPDH*) and ribosomal protein S18 (*RPS18*) as the endogenous controls, and their relative expressions, were processed using the 2^−ΔΔCt^ method, as described previously [23,24].

### 2.7. Western Blot

The total protein of the treated MAC-T cells was extracted with a whole protein extraction kit (KeyGEN, Nanjing, China), and its concentration was determined using the BCA protein assay kit (KeyGEN, Nanjing, China). The protein (20 μg) was separated from the samples using a 10% sodium dodecyl sulfate–polyacrylamide (SDS-PAGE) gel and transferred to a nitrocellulose (NC) membrane. Blocked with 5% skimmed milk for 2 h, the membrane was incubated for a whole night with primary antibody (1:500 dilution) at 4 °C. After that, we incubated the membrane with secondary antibody (1:5000 dilution) for 1 h at room temperature. In addition, a 5% BSA was used for blocking in the phosphorylated p65 (p-p65) assay. Finally, protein expression levels were detected using chemiluminescent ECL substrates (Millipore, Billerica, MA, USA). The β-actin was used as an internal control. The grayscale of the Western blot was quantified and analyzed using the Image J software (http://imagej.nih.gov/ij/ (accessed on 15 November 2021)).

All primary and secondary antibodies used were as follows: CD2AP (1:500, sc-25272), p-NF-κB p65 (1:500, sc-136548), AKT (1:500, sc-81434), and PI3K (1:500, sc-81434) were purchased from Santa Cruz Biotechnology, Inc. (Dallas, TX, USA). NF-κB p65 (1:500, AF0874), β-actin (1:500, AF7018), goat anti-mouse IgG-HRP (1:5000, S0002), and goat anti-rabbit IgG-HRP (1:5000, S0001) were purchased from Affinity Bioscience (Cincinnati, OH, USA).

### 2.8. ELISA

The bta-miR-199a-3p mimic, mimic NC, bta-miR-199a-3p inhibitor, or inhibitor NC were transfected into the MAC-T cells and induced by LPS, as described above (Section 2.3). The cell culture medium was centrifuged at 1000 rpm for 5 min at 4 °C, and the supernatant was collected. The OD values of the samples were measured at 450 nm using the appropriate ELISA kits (Table S3) according to the manufacturer’s instructions (CUSABIO, Wuhan, China), and the secretion levels of cytokines IL-1β, IL-6, and TNF-α in the MAC-T cell supernatant were calculated.

### 2.9. CCK-8 Assay

A CCK-8 kit (Meilunbio, Dalian, China) was used to analyze cell viability according to the manufacturer’s instructions. When the MAC-T cell density reached 60–70% in 96-well plates, the bta-miR-199a-3p mimic, mimic NC, bta-miR-199a-3p inhibitor, or inhibitor NC were transfected, and 50 ng/μL of LPS was added after 42 h to induce inflammation. After the cells were cultured for 5 h, 10 μL of a CCK-8 enhanced solution was added, and the absorbance was measured at 450 nm after incubation at 37 °C for 1 h. Cell-free wells were used as the blank.

### 2.10. Statistical Analysis

All data were displayed as mean and standard deviation (x ± s). The SPSS 25.0 software was used to test for significant differences in expression between the groups using independent sample *t*-test. A *p* < 0.05 value was used as the statistically significant threshold. Three biological replicates were performed per treatment.

## 3. Results

### 3.1. Bta-miR-199a-3p Was Up-Regulated in LPS-Induced MAC-T Cells

**Figure 1:**
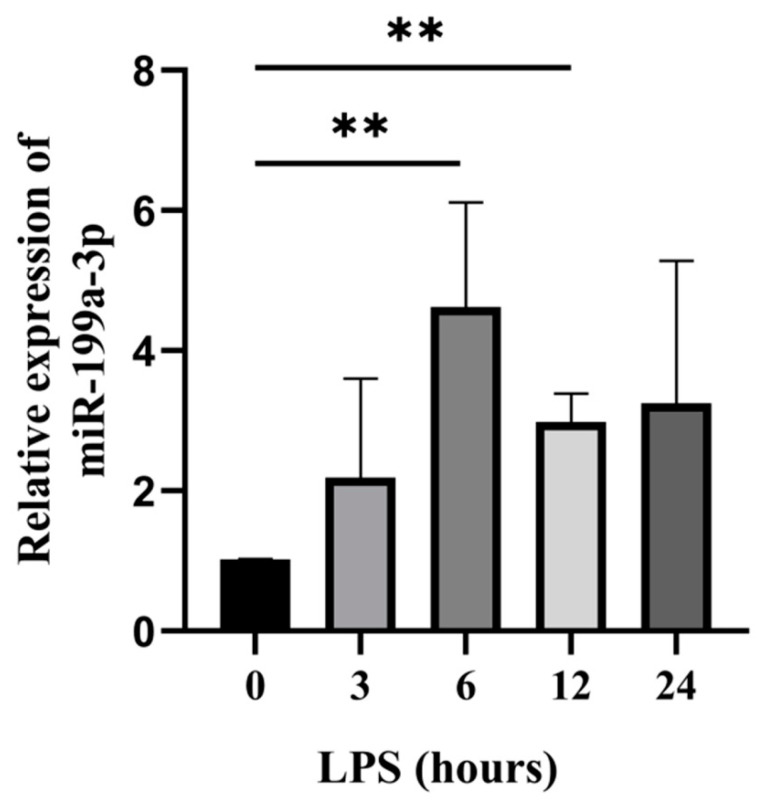
The expression of bta-miR-199a-3p was up-regulated in the LPS-induced MAC-T cells. The qRT-PCR analysis of bta-miR-199a-3p expression in the LPS-induced MAC-T cells at different times. The *GAPDH* and *RPS18* genes were used as the internal controls. ** refers to *p* < 0.01.

We used qRT-PCR to evaluate the expression of bta-miR-199a-3p at different induction times of LPS by establishing an LPS-induced MAC-T cell model. Compared to the cells at 0 h (control group), bta-miR-199a-3p expression was up-regulated at 3, 6, 12, and 24 h after LPS-induced inflammation. The expression at 6 h and 12 h showed an extremely significant increase (*p* < 0.01) (Figure 1). The results suggest that bta-miR-199a-3p might play an essential role in the inflammatory response of the MAC-T cells. In subsequent experiments, the LPS induction at 6 h was used to investigate the regulatory function of bta-miR-199a-3p, considering that the expression of the miRNA was the highest at this time.

### 3.2. Bta-miR-199a-3p Inhibited the Inflammatory Response of LPS-Induced MAC-T Cells

**Figure 2:**
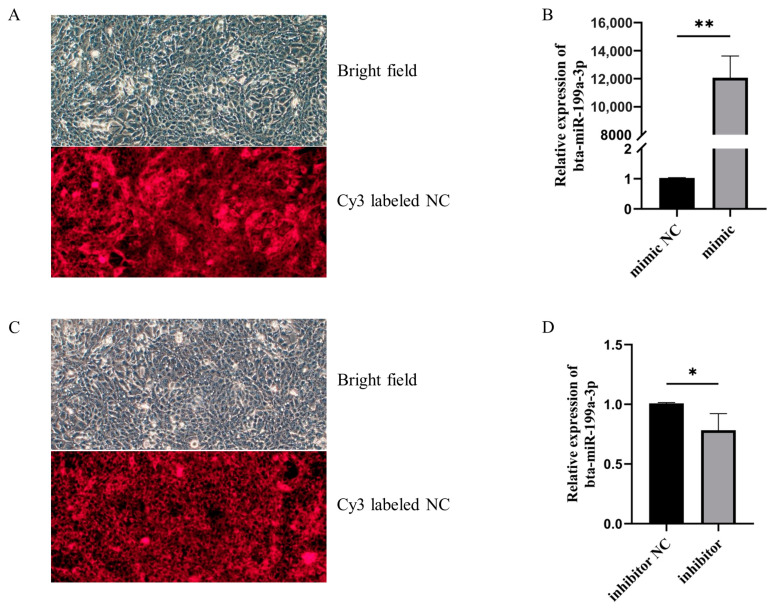
Transfection efficiency of bta-miR-199a-3p in the MAC-T cells. (**A**,**C**) The MAC-T cells were transfected with the bta-miR-199a-3p mimic (50 nM) or inhibitor (100 nM), and the transfection efficiency of the MAC-T cells was observed under an inverted fluorescence microscope. (**B**,**D**) The MAC-T cells were transfected with the miR-199a-3p mimic (50 nM) or inhibitor (100 nM), and the expression level of bta-miR-199a-3p was detected using qRT-PCR. *GAPDH* and *RPS18* were used as the internal controls. * refers to *p* < 0.05, ** refers to *p* < 0.01.

**Figure 3:**
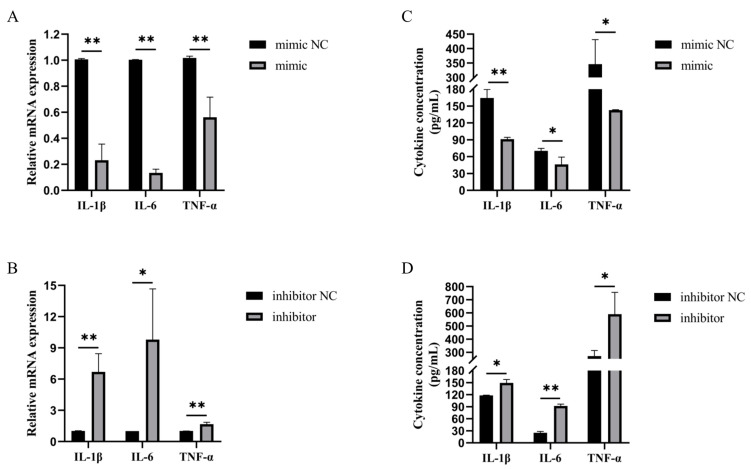
Bta-miR-199a-3p inhibits the production of inflammatory factors in the LPS-induced MAC-T cells. (**A**,**B**) The cells were transfected with the bta-miR-199a-3p mimic or inhibitor for 42 h and then induced with 50 ng/μL of LPS for 6 h, and qRT-PCR was used to detect the inflammatory cytokines IL-1β, IL-6, and TNF-α. The *GAPDH* and *RPS18* genes were used as the internal controls. (**C**,**D**) The protein levels of the cytokines IL-1β, IL-6, and TNF-α were determined using ELISA. * refers to *p* < 0.05, ** refers to *p* < 0.01.

LPS can promote the expression of inflammatory factors, such as IL-1β, IL-6, and TNF-α, thereby inducing an inflammatory response. To investigate the effect of bta-miR-199a-3p on inflammatory factors, we used the bta-miR-199a-3p mimic and bta-miR-199a-3p inhibitor to overexpress or silence the expression of the miRNA in the MAC-T cells treated with LPS. The transfected cells were observed for cy3-labeled mimic NC and inhibitor NC using an inverted fluorescence microscope and showed good transfection efficiency (Figure 2A,C). Detection of the expression of bta-miR-199a-3p using qRT-PCR showed favorable overexpression or silencing of bta-miR-199a-3p in the MAC-T cells, allowing for subsequent detection (Figure 2B,D). We measured the mRNA expression and secretion levels of these inflammatory cytokines in the MAC-T cells, and showed that an overexpression of bta-miR-199a-3p down-regulated the mRNA expression of IL-1β, IL-6, and TNF-α, and inhibited the secretion of IL-1β, IL-6, and TNF-α. However, the silencing of bta-miR-199a-3p up-regulated the expression of mRNA of the cytokines and promoted their secretion (Figure 3A–D). In conclusion, these results indicated that bta-miR-199a-3p could inhibit the LPS-induced inflammation in the MAC-T cells, but its mechanism required further study.

### 3.3. Effect of Bta-miR-199a-3p on the Proliferation of MAC-T Cells

**Figure 4:**
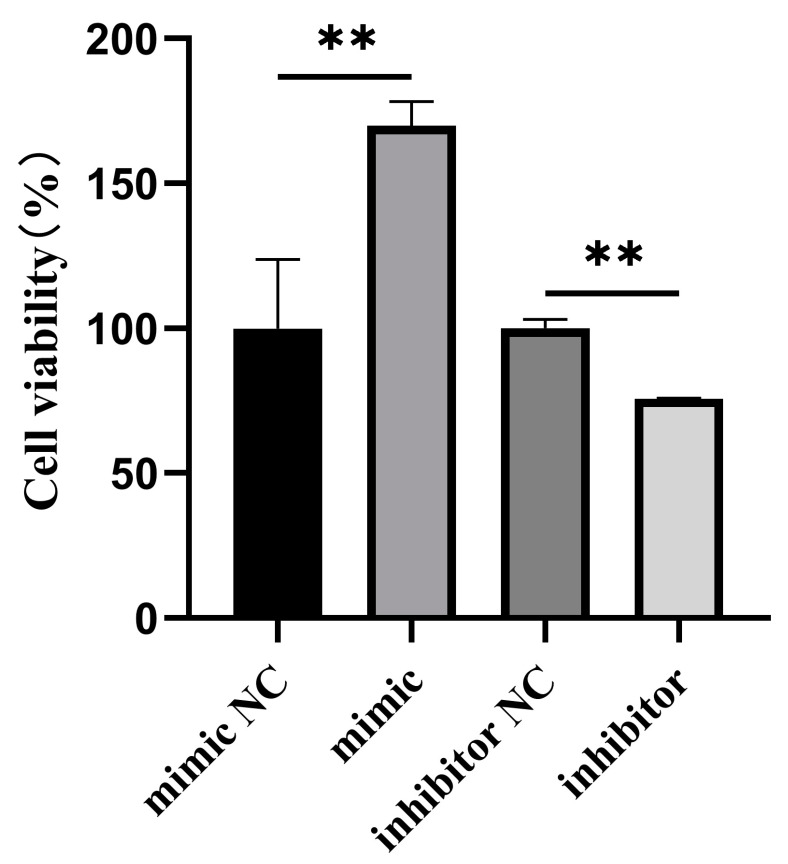
Effect of bta-miR-199a-3p on LPS-induced proliferation of MAC-T cells. The LPS-induced MAC-T cells’ viability was analyzed using the CCK-8 method after transfection with the bta-miR-199a-3p mimic or inhibitor, and the final data were normalized. ** refers to *p* < 0.01.

To detect the effect of bta-miR-199a-3p on the proliferation of MAC-T cells, overexpression or silencing of bta-miR-199a-3p were performed in the inflammatory MAC-T cells induced by LPS, while the CCK-8 method was used to detect cell proliferation (Figure 4). The results showed that bta-miR-199a-3p could promote the proliferation of the MAC-T cells during LPS-induced inflammation.

### 3.4. Bta-miR-199a-3p Inhibited the Expression of Apoptotic Genes

**Figure 5:**
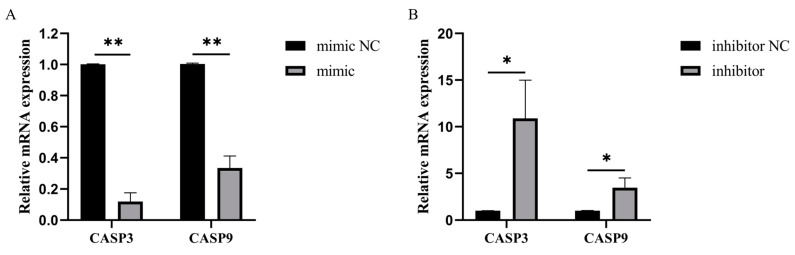
Effect of bta-miR-199a-3p on LPS-induced apoptosis gene expression in MAC-T cells. (**A**,**B**) After transfection with the bta-miR-199a-3p mimic or inhibitor, the mRNA expression levels of *CASP3* and *CASP9* in the LPS-treated MAC-T cells were detected using qRT-PCR. The *GAPDH* and *RPS18* genes were used as the internal controls. * refers to *p* < 0.05, ** refers to *p* < 0.01.

To explore the effect of bta-miR-199a-3p on LPS-induced apoptosis in MAC-T cells, we analyzed the changes in the mRNA levels of apoptotic genes by overexpression or silencing of bta-miR-199a-3p in the MAC-T cells. We found out that the overexpression of bta-miR-199a-3p significantly inhibited the expression of *CASP3* and *CASP9* genes (*p* < 0.01) (Figure 5A), while the inhibition of bta-miR-199a-3p promoted the expression of *CASP3* and *CASP9* genes (*p* < 0.05) (Figure 5B).

### 3.5. Bta-miR-199a-3p Does Not Directly Target MTOR

**Figure 6:**
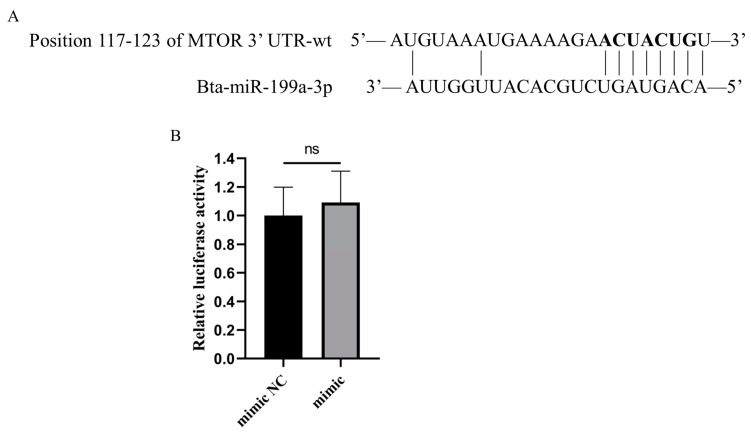
MTOR is not a molecular target of bta-miR-199a-3p. (**A**) TargetScan and miRWalk predicted the binding site of bta-miR-199a-3p to MTOR 3′-UTR. (**B**) The results of double luciferase activity detection. The miR-199a-3p mimic or mimic NC and the wild-type MTOR 3′-UTR luciferase reporter vector were co-transfected into 293T cells for double luciferase activity detection after 48 h. The relative luciferase activity on the ordinate represents the ratio of renilla luciferase activity to firefly luciferase activity. ns refers to *p* > 0.05.

MTOR has been identified as a target gene for human miR-199a-3p in previous studies [25,26], and was a potential target gene of bta-miR-199a-3p as predicted by two target gene prediction databases (Figure 6A). Therefore, we amplified and obtained bovine MTOR 3′-UTR containing a binding site for bta-miR-199a-3p and conducted a double luciferase reporter gene experiment. The relative luciferase activity results showed no significant difference between the mimic NC group and the bta-miR-199a-3p mimic group (*p* > 0.05) (Figure 6B), indicating that MTOR was not a target gene of bta-miR-199a-3p. We suggest that this result might be related to gene differences among different species.

### 3.6. CD2AP Is a Direct Molecular Target of Bta-miR-199a-3p

**Figure 7:**
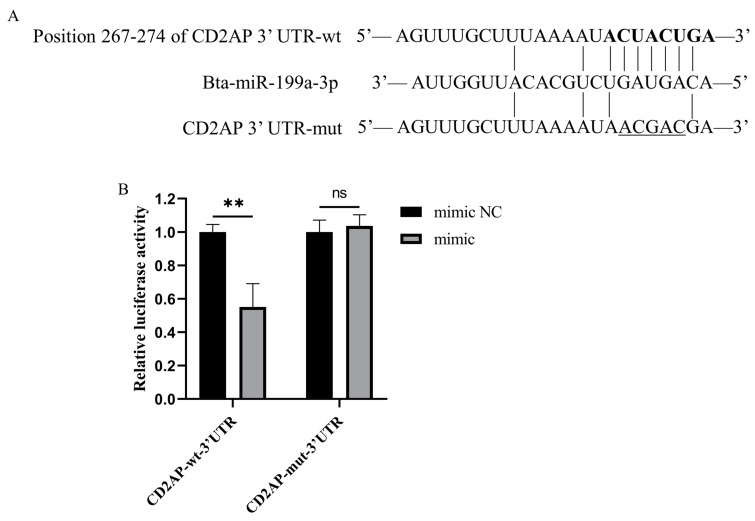
CD2AP is a molecular target of bta-miR-199a-3p. (**A**) TargetScan and miRWalk predicted the binding site of bta-miR-199a-3p to CD2AP 3′-UTR, where “ACGAC” is the mutated base. (**B**) The results of double luciferase activity detection. The miR-199a-3p mimic or mimic NC and the wild-type or mutation-type CD2AP 3′-UTR luciferase reporter vector were co-transfected into 293T cells for double luciferase activity detection after 48 h. The relative luciferase activity on the ordinate represents the ratio of renilla luciferase activity to firefly luciferase activity. ** refers to *p* < 0.01, ns refers to *p* > 0.05.

The target gene prediction indicated that CD2AP might be a potential target gene of bta-miR-199a-3p (Figure 7A). Therefore, we performed a double luciferase reporter gene experiment to identify their targeted regulation relationship. The results showed that the luciferase activity of the bta-miR-199a-3p mimic+3′UTR-CHECK^TM^-2-wt transfection group was significantly lower than that of the mimic NC+3′UTR-CHECK^TM^-2-wt transfection group (*p* < 0.01), indicating that the miRNA could directly target the 3′-UTR of CD2AP mRNA. However, there was no notable change in the 3′UTR-CHECK^TM^-2-mut group (Figure 7B), validating the accuracy of the binding site.

### 3.7. Bta-miR-199a-3p Negatively Regulates the Expression of CD2AP mRNA and Protein in MAC-T Cells

**Figure 8:**
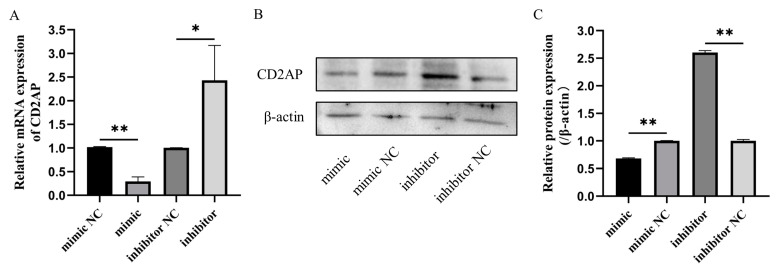
Bta-miR-199a-3p negatively regulates the mRNA and protein expression of CD2AP in the MAC-T cells. (**A**) Effect of overexpression or inhibition of bta-miR-199a-3p on CD2AP mRNA expression. (**B**,**C**) Effect of overexpression or inhibition of bta-miR-199a-3p on CD2AP protein expression: (**B**) is the result of Western blot, and (**C**) is the image analysis result of (**B**) (with β-actin as the standardized correction). * refers to *p* < 0.05, ** refers to *p* < 0.01.

To further determine whether bta-miR-199a-3p in the MAC-T cells had a regulatory effect on CD2AP, the qRT-PCR and Western blot methods were applied to detect the expression of CD2AP in the cells after bta-miR-199a-3p overexpression and silencing. The results indicated that bta-miR-199a-3p negatively regulated the mRNA and protein levels of CD2AP in the MAC-T cells (Figure 8A–C). Combined with the double luciferase reporter gene results, we identified CD2AP as a molecular target of bta-miR-199a-3p in the MAC-T cells.

### 3.8. Bta-miR-199a-3p Inhibited LPS-Induced Activation of the PI3K/AKT/NF-κB Pathway

**Figure 9:**
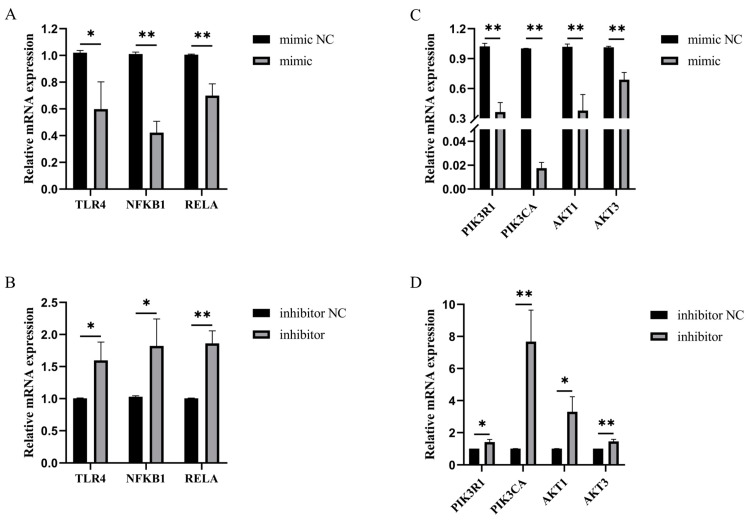

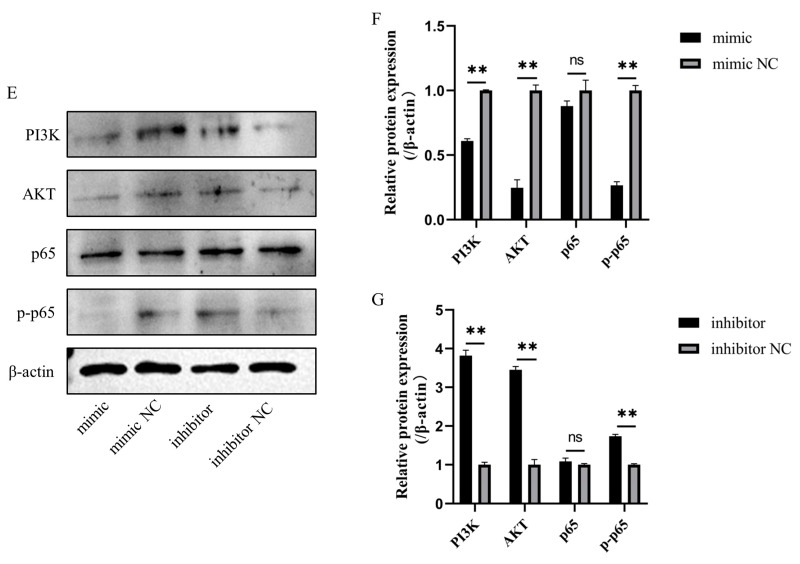
Effects of bta-miR-199a-3p on the LPS-induced PI3K/AKT/NF-κB pathway. (**A**,**B**) Effects of overexpression or inhibition of bta-miR-199a-3p on the mRNA expression levels of TLR4, NFKB1, and RELA. (**C**,**D**) Effects of overexpression or inhibition of bta-miR-199a-3p on the mRNA expression levels of PIK3R1, PIK3CA, AKT1, and AKT3. (**E**–**G**) Effects of overexpression or inhibition of bta-miR-199a-3p on the protein expression levels of PI3K, AKT, p65, and p-p65: (**E**) is the result of Western blot, and (**F**,**G**) are the image analysis results of (**E**) (with β-actin as a standardized correction). * refers to *p* < 0.05, ** refers to *p* < 0.01, ns refers to *p* > 0.05.

The overexpression of bta-miR-199a-3p inhibited the LPS-induced mRNA expression of TLR4, NFKB1 (p50), RELA (p65), AKT1, AKT3, PIK3CA, and PIK3R1 in the MAC-T cells, compared to the mimic NC group (Figure 9A,C). The silencing of bta-miR-199a-3p produced the opposite results (Figure 9B,C), suggesting that bta-miR-199a-3p might inhibit LPS-induced activation of the PI3K/AKT/NF-κB pathway. Western blot experiments were performed to further verify the above inference, confirming that the overexpression of bta-miR-199a-3p inhibited the expression of PI3K, AKT, and p-p65 proteins (Figure 9E,F). However, the silencing of bta-miR-199a-3p promoted the expression of the proteins (Figure 9E,G). In summary, all the above results indicate that bta-miR-199a-3p inhibits the activation of the PI3K/AKT/NF-κB signaling pathway in LPS-induced inflammation in MAC-T cells.

## 4. Discussion

Mastitis is an inflammatory process leading to pathological changes in dairy cow mammary gland tissue, which can cause damage to bMECs [27]. In recent years, the treatment of cow mastitis has been improved to a certain extent, but its incidence remains high. In the absence of effective treatment, infected dairy cows are eliminated, causing economic losses to farmers [28]. Therefore, it is imperative to determine the inflammatory damage mechanism of dairy cow mastitis to help reduce the incidence of the disease. In our experiment, we showed that bta-miR-199a-3p had an effect on LPS-induced MAC-T cells. 

Many studies have shown that miRNAs are important regulators of inflammation since they play an essential role in cell proliferation and apoptosis [29]. miR-199a-3p is a member of the miRNA family, which is highly conservative in different mammals, and plays a regulatory role in inflammation [19]. However, the biological function of bta-miR-199a-3p in LPS-induced inflammation of bMECs is still unclear and is only mentioned in the literature related to miRNA expression profiling [30]. Luoreng et al. [30] found that bta-miR-199a-3p was up-regulated in mammary tissue of *E. coli*-induced dairy cow mastitis. This study found that the expression of bta-miR-199a-3p was up-regulated in inflammatory MAC-T cells, which was consistent with the sequencing results in LPS-induced mammary gland inflammation in dairy cows, suggesting that the miRNA played a crucial role in cow mastitis. Therefore, we further explored its other functions.

The toll-like receptors (TLRs) play a vital role in recognizing a variety of microorganisms and activating the innate immune system. The TLRs and their ligands are significant in various pathological conditions, such as inflammation, tumorigenesis, and autoimmune disorders [31,32]. Among them, TLR4 can recognize pathogen-associated molecular patterns (such as LPS) on the surface of Gram-negative bacteria before activating the downstream signaling pathways to mediate the production of IL-1β, IL-6, TNF-α, and other inflammatory factors [33]. To investigate the regulatory effect of bta-miR-199a-3p on bMEC inflammation, the effects of overexpression and silencing of bta-miR-199a-3p on inflammatory factors were detected using qRT-PCR and ELISA. The overexpression of bta-miR-199a-3p significantly reduced the LPS-induced production of IL-1β, IL-6, and TNF-α in the MAC-T cells, whereas the silencing of the miRNA yielded more cytokines. Bta-miR-199a-3p inhibited MAC-T cells’ inflammatory response. It has been confirmed that miR-199a-3p can activate the PI3K/AKT signaling pathway and, thus, can regulate cell proliferation and apoptosis [34,35]. The expression of key apoptotic genes was detected after the overexpression and silencing of bta-miR-199a-3p, and the proliferation of the LPS-induced MAC-T cells was detected using the CCK-8 method. Our results showed that bta-miR-199a-3p could promote the proliferation of MAC-T cells under inflammatory conditions.

CD2AP is a vital adapter protein that is widely expressed in immune cells, epithelial cells, and neurons at a high level [36,37]. CD2AP is closely related to proteinuria since it participates in cellular immunity, cytoskeletal assembly, maintenance of the Hiatal septum structure, and signal transduction [38]. The protein also maintains the physiological integrity of the glomerular filtration barrier [38]. Most studies on CD2AP have focused on kidney and Alzheimer’s diseases [37]. However, the mechanisms of action of CD2AP in other inflammations are rarely reported. In the present study, the double luciferase reporter gene detection verified that bta-miR-199a-3p targeted the gene’s 3′-UTR and significantly inhibited the expression levels of the bovine CD2AP mRNA and protein. CD2AP binds to a subunit of PI3K that together stimulate PI3K-dependent activation of AKT [39]. One study has shown that hepatitis C virus-infected cells have higher levels of CD2AP, which promotes hepatitis C virus transmission and steatosis by disrupting insulin signaling [40]. In human, miR-133a-3p overexpression inhibits the inflammatory response and apoptosis of bronchial epithelial cells infected with a respiratory syncytial virus by negatively regulating the expression of CD2AP [41]. This reduces cell damage and interferes with human CD2AP to inhibit the expression of TNF-α, IL-6, and IL-1α and cell apoptosis [41]. Umbelliferone inhibits the expression of CD2AP and the activation of the TLR/NF-κB pathway, reducing the secretion of inflammatory factors in a rat model of diabetic nephropathy, thereby improving renal function [42]. Inhibition of human miR-188-5p activates the PI3K/AKT/mTOR signaling pathway, subsequently inhibiting cell proliferation and promoting apoptosis [43]. However, inhibition of CD2AP reverses this process [38]. These studies are similar to our results.

The NF-κB signaling pathway found in the PI3K/AKT downstream signaling pathway of inflammation plays a central role in transcriptional regulation and is stimulated by many cells [39]. This signaling pathway participates in the expression and regulation of various genes and plays a key regulatory role in inflammation [44]. Multiple miRNAs also regulate inflammation through the PI3K/AKT/NF-κB signaling pathway [45]. Among them, p65 is the key protein at the end of this pathway. The amount of nuclear translocation in the activated state of p-p65 reflects the developmental state of inflammation, while its reduced expression indicates the inhibition of NF-κB signaling pathway activation, thereby attenuating the inflammatory response [46]. Interestingly, the overexpression of bta-miR-199a-3p in the LPS-induced MAC-T cells could inhibit the expression of key genes and proteins in the PI3K/AKT/NF-κB signaling pathway, while the silencing of bta-miR-199a-3p promoted their expression. These results indicate that bta-miR-199a-3p could inhibit LPS-induced apoptosis and inflammatory response in MAC-T cells by inhibiting the PI3K/Akt/NF-κB signaling pathway.

## 5. Conclusions

In summary, bta-miR-199a-3p plays a significant role in cellular inflammatory and immune responses. We found that the up-regulation of bta-miR-199a-3p in inflammatory MAC-T cells regulates the PI3K/AKT/NF-κB signaling pathway by inhibiting CD2AP, which subsequently inhibits bMEC inflammation and apoptosis. This study showed the regulatory mechanism of bta-miR-199a-3p in the immune response to bMEC infection, which may provide a potential target for mastitis treatment.

## Data Availability

Not applicable.

## Supplementary Materials

The following supporting information can be downloaded at https://www.mdpi.com/article/10.3390/cells11213518/s1, Figure S1: Identification of the MAC-T cells using immunofluorescence with Cytokeratin 18 (100×); Table S1: List of primers for 3′-UTR amplification of the candidate target genes; Table S2: List of all primer sequences using qRT-PCR; Table S3: The information of the ELISA kit.
