# Supplementary material for: Bta-miR-199a-3p Inhibits LPS-Induced Inflammation in Bovine Mammary Epithelial Cells via the PI3K/AKT/NF-κB Signaling Pathway
Source: Cells. 2022 Nov 7;11(21):3518. doi: 10.3390/cells11213518 (PMC9656885; doi:10.3390/cells11213518)
Supplement: Supplementary file 1 [file cells-11-03518-s001.zip › Figure S1. Identification of the MAC-T cells using immunofluorescence with Cytokeratin 18 (100í┴)..pdf]

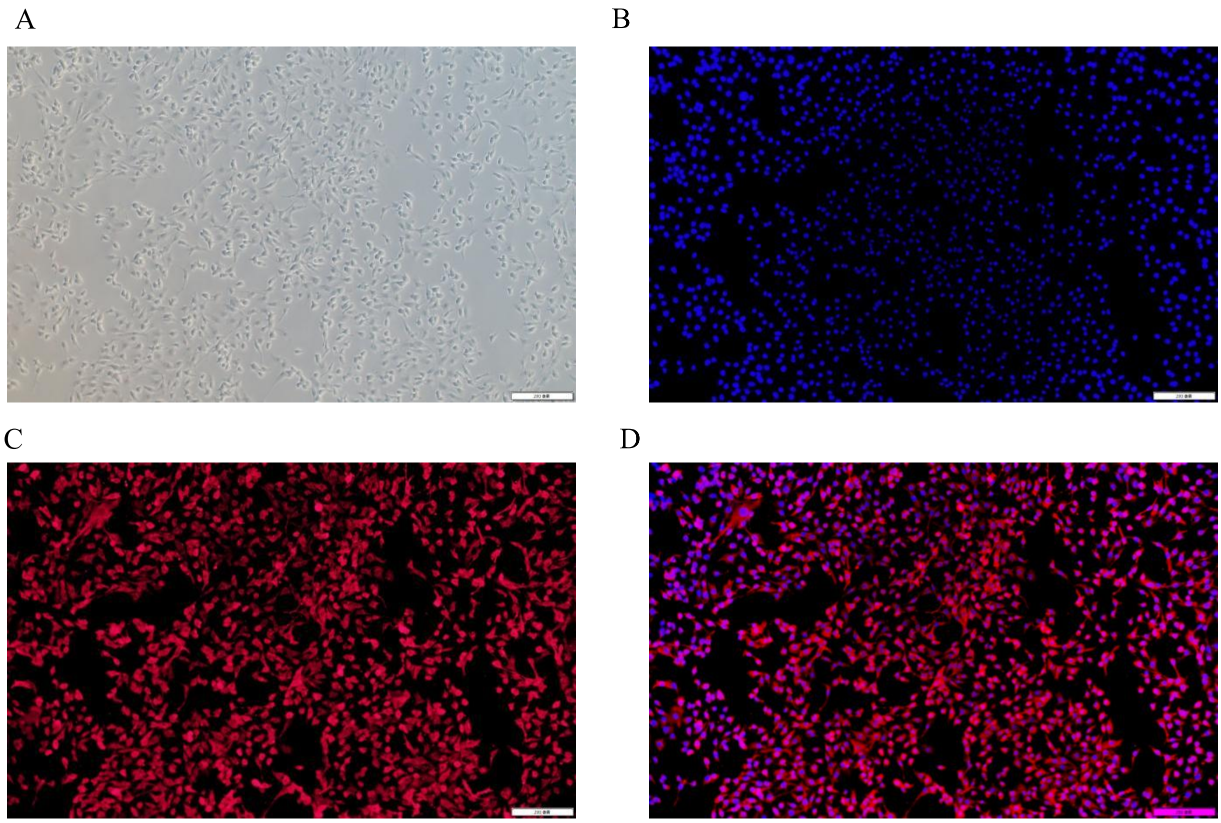

Figure S1. Identification of the MAC-T cells using immunofluorescence with Cytokeratin 18 (100×). (A) Bright field. (B) DAPI staining. (C) Immunofluorescence with Cytokeratin 18 labeled with Cy3. (D) Merge of B and C.
